# Associations of specific phobia and its subtypes with physical diseases: an adult community study

**DOI:** 10.1186/s12888-016-0863-0

**Published:** 2016-05-21

**Authors:** Cornelia Witthauer, Vladeta Ajdacic-Gross, Andrea Hans Meyer, Peter Vollenweider, Gerard Waeber, Martin Preisig, Roselind Lieb

**Affiliations:** Department of Psychology, Division of Clinical Psychology and Epidemiology, University of Basel, Basel, Switzerland; Department of Psychiatry, Psychotherapy, and Psychosomatics, University Hospital of Psychiatry, University of Zurich, Zurich, Switzerland; ZInEP, The Zurich Program for Sustainable Development of Mental Health Services, University of Zurich, Zurich, Switzerland; Department of Medicine, Internal Medicine, University Hospital of Lausanne, Lausanne, Switzerland; Department of Psychiatry, University Hospital of Lausanne, Lausanne, Switzerland

**Keywords:** Specific phobia, Comorbidity, Physical diseases, Representative survey

## Abstract

**Background:**

Specific phobia is the most prevalent anxiety disorder in the community and is associated with substantial impairment. Comorbidity with physical diseases is assumed and has important implications for etiology, treatment, or prevention of the comorbid conditions. However, due to methodological issues data are limited and subtypes of specific phobia have not been investigated yet. We examined the association of specific phobia and its subtypes with physical diseases in a representative community sample with physician-diagnosed physical diseases and diagnostic criteria of specific phobia.

**Methods:**

Data of the German Mental Health Survey from 4181 subjects aged 18–65 years were used. Specific phobia was diagnosed using M-CIDI/DIA-X interview; physical diseases were assessed through a self-report questionnaire and a medical interview. Logistic regression analyses adjusted for sex were calculated.

**Results:**

Specific phobia was associated with cardiac diseases, gastrointestinal diseases, respiratory diseases, arthritic conditions, migraine, and thyroid diseases (odds ratios between 1.49 and 2.53). Among the subtypes, different patterns of associations with physical diseases were established. The findings were partially replicated in the Swiss PsyCoLaus Study.

**Conclusions:**

Our analyses show that subjects with specific phobia have an increased probability for specific physical diseases. From these analyses etiological mechanisms of specific phobia and physical disease can be deduced. As subtypes differed in their patterns of associations with physical diseases, different etiological mechanisms may play a role. The findings are highly relevant for public health in terms of prevention and therapy of the comorbid conditions.

## Background

Specific phobia is the most prevalent anxiety disorder in the community [1, 2]. It is associated with significant impairment and distress [3] and with a loss of work days [4]. Further, specific phobia is a predictor for increased suicidal tendency [5] and it is thought to be a risk factor for the later development of other mental disorders such as major depression [6, 7] or anxiety disorders [8]. Additionally, specific phobia is highly comorbid with other mental disorders, especially with anxiety disorders and mood disorders [9, 10].

Research has suggested that besides being comorbid with other mental disorders, specific phobia may be highly comorbid with physical diseases, too. Having any anxiety disorder during the last year has been associated with neurological, vascular, respiratory, gastrointestinal, metabolic, bone, and infectious diseases [11–14]. Further, a longitudinal analysis showed that self-reported gastrointestinal disease predicted the onset of specific phobia in older adults during the next three years [15]. Subjects with coronary heart disease reported elevated levels of phobic anxiety [16] and phobic anxiety is associated with cardiac mortality [17]. Among men with high levels of phobic anxiety, an increased prevalence rate of Parkinson’s disease was found compared to men with low levels of phobic anxiety [18]. Further, a review revealed that subjects with chronic obstructive pulmonary disease reported a prevalence of 10–17 % of specific phobia [19]. Community studies in addition established an association between specific phobia and migraine [20], respiratory diseases [11], ulcer [21], vascular diseases [22] and heart diseases [23].

Most research has suggested a link between specific phobia and physical diseases. However, there are methodological issues that limit the generalizability of the findings. First, not all studies used *Diagnostic and Statistical Manual of Mental Disorders (DSM)* [24] or *International Classification of Diseases* (*ICD*) [25] diagnostic criteria of specific phobia. Second, the physical diseases were mainly assessed by self-report rather than diagnosed by a physician. Third, most studies focused on one specific physical disease and therefore provide limited information on the association with different physical diseases.

Knowledge of the comorbidity of specific phobia and physical diseases could influence research on the etiology of both physical diseases and specific phobia through the detection of possible etiological mechanisms. It is also highly relevant for public health because some studies have suggested that anxiety can complicate the treatment of chronic medical diseases and may therefore be associated with worse treatment outcomes [26, 27].

DSM-IV differentiates among five specific phobia subtypes (animal type, natural type, blood-injection-injury type, situational type and other type), which primarily differ with respect to content of subjective fear [28]. Variation with respect to age of onset among the subtypes has also been observed [29]. Further, some studies established differences among the comorbidity patterns with other mental disorders [28, 30, 31]: Youths with specific phobias of the natural type reported more depressive symptoms and showed higher prevalence rates of other anxiety disorders than youths with animal phobias [32]; situational phobia was associated with more panic attacks than other specific phobia subtypes [31]; and individuals with blood-injection phobia had a higher prevalence of marijuana abuse, depression, panic disorder, obsessive-compulsive disorder, or social phobia compared to individuals without blood-injection phobia [31, 33, 34]. Additionally, subjects with specific phobia of the natural type reported more somatic symptoms than subjects with animal phobia [32].

As for the different associations with mental disorders, subtypes of specific phobia may also show different comorbidity patterns with physical diseases. Psychobiological aspects might help to explain why these differences may be expected: There is evidence suggesting that different physiological processes in some biological systems may be involved in the different fear reaction of specific phobia subtypes [29]. Subjects with animal phobia show triggered elevations in heart rate, blood pressure, norepinephrine, and epinephrine when they are exposed to the phobic stimulus. Subjects with blood-injection phobia on the other hand show an initial rise in heart rate and blood pressure followed by vasovagal fainting characterized by bradycardia and hypotension [31, 33]. Further, some evidence suggests partially distinct neurobiological substrates of blood-injection and animal phobia [35, 36]. A study investigating the neural response during the presentation of phobia-specific stimuli established that subjects with animal phobia showed activation in dorsal anterior cingulate and anterior insula, in comparison to subjects with Blood Injection Phobia showing activation in the thalamus and visual/attention areas [35].

Based on these empirical findings, we suggest that specific phobia subtypes are associated with different physical diseases that may be linked to distinct physiological processes. Analyses addressing similarities and differences among specific phobia subtypes are important to elucidate potential etiological pathways. However, to the best of our knowledge no study has evaluated the association between specific phobia subtypes and physical diseases in the community.

### Hypotheses

The aim of our study therefore was to analyze the association of *DSM-IV* specific phobia and its subtypes and a broad range of physical diseases in the German Health Interview and Examination Survey, Mental Health Supplement (GHS-MHS). We addressed the methodological limitations reported above by using a wide range of physician-diagnosed physical diseases and specific phobia assessed by a well-validated structured interview. Further, we aim to replicate the findings in a separate data set, the baseline investigation of the PsyCoLaus Study.

Based on the established associations between specific phobia and physical diseases in community samples as presented above, we specifically hypothesized for our community sample that

1. Having any specific phobia is associated with migraine
2. Having any specific phobia is associated with respiratory diseases
3. Having any specific phobia is associated with gastrointestinal diseases
4. Having any specific phobia is associated with cardiac diseases
5. Having any specific phobia is associated with vascular diseases

As there is not enough empirical knowledge to formulate specific hypotheses concerning the associations between subtypes of specific phobia and physical diseases, we further investigated these associations in an explorative manner.

## Methods

### Design and sample of the GHS-MHS

The GHS-MHS, conducted in 1997, was the first nationwide cross-sectional study for medical and social assessments in Germany. The GHS-MHS was commissioned by the German Ministry of Science, Research and Education and approved by the relevant institutional review board of the Robert Koch Institute (Berlin, Germany). The aim of the core study was the assessment of sociodemographic characteristics, physical diseases, impairments, and health-care utilization in a representative community sample of 7,124 subjects ages 18–79 years (overall response rate 61.5 %). The sample was stratified and randomized from 113 communities throughout Germany with 130 sampling units (step 1: selection of communities, step 2: selection of sampling units, step 3: selection of inhabitants) [2, 37]. The data were weighted and confidence intervals were calculated by the Huber–White sandwich method to account for the weighting scheme as well as the stratified sampling design [37].

Mental disorders were assessed in a two-stage design: The first stage entailed the administration of a 12-item screening questionnaire for mental disorders at the end of the medical examination of the core survey (the Composite International Diagnostic Screener) [38]. The second stage involved the administration of a structured psychopathological interview, the Munich Composite International Diagnostic Interview (DIA-X/M-CIDI) to all core survey respondents who had been screened positive for a mental disorder and to a random sample of 50 % who screened negative [38]. This subsample of the GHS is the sample of the Mental Health Supplement and included 4181 subjects aged 18–65 years. The M-CIDI interview was completed by 87.6 % of the subjects (conditional response rate). All subjects gave their written informed consent. Further descriptions of aims, design, and methods as well as sociodemographic characteristics of the whole GHS-MHS sample can be found elsewhere [37].

### Specific phobia in the GHS-MHS

The fully structured DIA-X/M-CIDI interview was used for the diagnostic assessments in the GHS-MHS [39] covering both *DSM-IV* and *ICD-10* criteria. Through the structured interview symptoms, syndromes, onset, duration and severity were assessed. Trained psychologists and physicians conducted the interview [37]. The DIA-X/M-CIDI diagnostic algorithms were used to obtain the diagnostic findings reported in this paper [40]. There was substantial test–retest reliability (kappa values between 0.56 and 0.81) [41]; the sensitivity of the DIA-X/M-CIDI diagnoses ranges from 87.5 % to 100 %, their specificity from 71.2 % to 100 % [42]. The validity of the full diagnoses ranges from moderate to excellent when compared to diagnoses made by independent treating physicians in a sample of randomly chosen patients [42].

We used the 12-month *DSM-IV* diagnosis of specific phobia. Additionally, the 12-month diagnoses of the following subtypes of specific phobia were used: the animal subtype (referring to fear related to insects, snakes, birds, or other animals), the natural subtype (referring to fear related to height, storm, water), the blood-injection subtype (referring to fear related to seeing blood, injection, going to the dentist or hospital), the situational subtype (referring to flying in a plane, being in a small closed room, in a cellar, tunnel, or elevator), and the other subtype (referring to any other specific fear not matching any other subtype).

### Physical diseases in the GHS-MHS

Physical diseases were assessed by a self-report questionnaire and a standardized computer-assisted medical interview by a general practice physician. Using the information on physical diseases in the self-report questionnaire, the physician collected data on age of onset, lifetime prevalence, 12-month prevalence, and point prevalence (4 weeks) of 44 physical diseases [37]. Blood pressure and anthropometric measurements were conducted and blood and urine samples were collected. Diagnoses were then supplemented and revised based on these laboratory analyses [37]. The present analyses are based on the physicians’ diagnoses during the medical interview.

### Replication study

#### Design and sample of the PsyCoLaus Study

As a replication study, we used the baseline investigation of the CoLaus/PsyCoLaus cohort study [43, 44]. CoLaus/PsyCoLaus conducted in Lausanne, Switzerland, was designed to study mental disorders and cardiovascular risk factors in the general population. The subjects aged 35–75 years were randomly selected through the population register of the city of Lausanne. All participants of the somatic investigation (CoLaus) aged between 35 and 66 years were asked to also undergo a comprehensive psychiatric evaluation (PsyCoLaus), resulting in a subsample of 3720 subjects (response rate: 67 %) [44]. The psychiatric assessment took place between 2004 and 2008.

### Specific phobia in the PsyCoLaus Study

The psychiatric evaluation in PsyCoLaus was based on the French version of the semi-structured Diagnostic Interview for Genetic Studies (DIGS; [45, 46]), which elicits DSM-IV axis I criteria and suicidal behavior and extensive information on the course and chronology of comorbid conditions. As the phobia section of the original DIGS interview was brief [46] it was replaced by the chapter from the semi-structured Schedule for Affective Disorders and Schizophrenia – Lifetime Version (SADS-LA) [47, 48]. The semi-structured interview revealed excellent inter-rater and fair to good test-retest reliability for major mood and psychotic disorders [49, 50]. Regarding anxiety disorders, applying the French translation of the SADS-LA, Leboyer et al. [46] found satisfactory test-retest reliability (mean interval 3.2 months) for panic disorder/agoraphobia (Yule’s Y = 0.43), Generalized Anxiety Disorder (GAD) (Yule’s Y = 0.61) and phobic disorders (Yule’s Y = 0.66). The Yule coefficient for the overall category of anxiety disorders was 0.49. Our own reliability study [50], based on a sample of 136 patients who also completed the combined DIGS – SADS-LA section for specific anxiety disorders, revealed perfect inter-rater agreement for all specific anxiety disorders except for agoraphobia (Yule’s Y = 0.96). The Yule’s Y coefficients for the 6-week test-retest reliability were 0.58 for panic disorder, 0.55 for agoraphobia, 0.44 for social phobia, 0.77 for specific phobia and 0.64 for OCD. Regarding GAD, no test-retest agreement was obtained.

During the interview, the subjects were asked if they had experienced symptoms of anxiety during certain situations related to the *DSM-IV* subtypes of specific phobia during their lifetime (e.g. “Did you feel anxious when you were flying in a plane?” or “Did you feel anxious when you were facing a certain animal?”). Subtypes were then derived from the information collected during the interview. For our analyses, the following subtypes were created based on *DSM-IV* criteria (comparable to those of the GHS-MHS): animal, natural, situational, blood-injection and the other subtype. As the ages of onset and offset of each disorder were assessed, 12 month prevalence of specific phobia and its subtypes could be established.

### Physical diseases in the PsyCoLaus Study

Cardiovascular and metabolic diseases were assessed during the physical CoLaus evaluation through a medical interview, physical examinations and blood and urine tests. Moreover, in the medical part of the DIGS interview during the psychiatric evaluation information was collected on additional physical lifetime diseases. During this evaluation age at onset information for physical diseases were also collected.

For the present analyses, we used data from the CoLaus and PsyCoLaus Studies. Cardiac diseases and vascular diseases were diagnosed during the CoLaus Study by an adjudication committee according to the latest diagnostic criteria, and diagnoses of hypertension and diabetes were based on measurements of blood pressure and fasting glucose [43]. For the other groups of diseases data stemmed from the medical part of the psychiatric interview. To enable comparison with the GHS-MHS, the same groups of physical diseases were built. However, in the groups of cardiac diseases, arthritic conditions and vascular diseases not all or different physical diseases were assessed among these groups in both studies (as indicated in Table 1).

**Table 1:** Frequency of specific phobia and its subtypes and physical diseases in the GHS-MHS (12 months) and the PsyCoLaus Study (12 months for specific phobia and lifetime for physical diseases)

Phobia/disease	GHS-MHS		PsyCoLaus	
	No.	%	No.	%
Any specific phobia^a^	388	7.6	518	13.9
Subtypes^b^				
Animal subtype	68	1.5	112	3.0
Natural subtype	75	1.4	37	1.0
Blood-injection subtype	64	1.5	18	0.5
Situational subtype	89	1.8	48	1.3
Other subtype	3	0.08	10	0.3
Hypertension	581	13.1	1110	29.8
Cardiac diseases (heart circulation disturbances, narrowing of the coronary vessels, angina pectoris, cardiac infarct, heart weakness, heart insufficiency)^c^	100	2.2	199	5.3
Respiratory diseases (asthma, chronic bronchitis)	284	7.0	572	15.3
Gastrointestinal diseases (ulcer, gastritis)	268	6.3	252	6.7
Diabetes (with or without insulin treatment)	115	2.7	194	5.2
Arthritic conditions (wear and tear type, inflammatory diseases of the joints)^d^	1107	25.9	773	20.8
Allergies (hay fever, allergic eczema, allergic hives, neurodermatitis, food allergy, allergic conjunctivitis)	747	18.1	1018	27.3
Migraine headaches	491	10.3	562	15.2
Neurological diseases (epilepsy, Parkinson’s disease, multiple sclerosis)	27	0.5	94	2.5
Thyroid diseases	445	10.0	164	4.4
Vascular diseases (stroke, brain circulation disturbance, leg circulation disturbance, artery occlusion, varicose veins, vein thrombosis)^e^	536	12.4	303	8.2

*Note.* No: Unweighted number of subjects; %: Percentage (in the GHS-MHS: weighted percentage), *GHS-MHS* German Health Survey, Mental Health Supplement ^a^Including animal, natural, blood-injection, situational, or any other subtype. ^b^subjects did not fulfill the criteria of any other subtype. ^c^In the PsyCoLaus Study: coronary artery disease, angina, myocardial infarct, percutaneous coronary intervention, coronary artery bypass graft, pacing, heart failure, valvular heart disease, cardiomyopathy. ^d^In the PsyCoLaus Study: rheumatoid arthritis, osteoarthritis, arthritis. ^e^In the PsyCoLaus Study: arrhythmia, stroke, peripheral artery disease

### Ethics, consent and permissions

All subjects of the GHS-MHS and the PsyCoLaus Study gave their written informed consent. The GHS-MHS was commissioned by the German Ministry of Science, Research and Education and approved by the relevant institutional review board of the Robert Koch Institute (Berlin, Germany). The Ethics Committee of the University of Lausanne approved both the physical (CoLaus) and psychiatric (PsyCoLaus Study) evaluation. All subjects gave their written informed consent [44].

### Statistical analyses

Logistic regression analyses were used to evaluate the association of specific phobia and physical diseases in both the GHS-MHS and the PsyCoLaus Study (specific phobia as predictor, physical diseases as outcome). As specific phobia and physical diseases were both associated with sex, the associations between the two were controlled for sex. To further investigate the impact of sex on the results, we checked the interactions of sex and specific phobia on the association with physical diseases. Additionally, using the age of onset data available for both specific phobia and physical diseases, we evaluated the temporal order of onset of comorbid cases. We considered a *p* value <0.05 as statistically significant. The analyses in the GHS-MHS study were carried out using STATA 11.0 [51]. The analyses in the PsyCoLaus Study were carried out using SPSS version 20 [52].

## Results

### The 12-month prevalence of specific phobia and physical diseases in the GHS-MHS

In the GHS-MHS, the 12-month prevalence of specific phobia was 7.6 %, as shown in Table 1. The most prevalent subtype of specific phobia was the situational subtype (1.8 %), and the other subtype was the least prevalent (0.08 %). The most prevalent physical diseases were arthritic conditions with a 12-month prevalence of 25.9 %, followed by allergies (18.1 %). The least prevalent physical diseases were neurological diseases (0.5 %).

### Association between specific phobia and physical diseases in the GHS-MHS (hypotheses 1–5)

As shown in Table 2, having any specific phobia was associated with cardiac diseases, gastrointestinal diseases, respiratory diseases, arthritic conditions, migraine, and thyroid diseases (odds ratios [ORs] ranging between 1.49, 95 % CI [1.15–1.92], for arthritic conditions and 2.53, CI [1.73–3.69], for gastrointestinal diseases). Additionally, having any specific phobia was associated with any physical disease (OR = 1.87, [1.30–2.68]). Therefore Hypotheses 1–4 could be confirmed, whereas Hypothesis 5 had to be rejected as no association between any specific phobia and vascular diseases could be proved.

**Table 2:** Odds ratios and confidence intervals of physical diseases (12 months) for specific phobia and its subtypes (12 months) compared to a reference group that had no specific phobia during the past 12 months in the German Health Interview and Examination Survey – Mental Health Supplement (*N* = 4,181)

Phobia	Physical disease											
	Hypertension (*n* = 581)	Cardiac diseases (*n* = 100)	Gastro-intestinal diseases (*n* = 268)	Respiratory diseases (*n* = 284)	Diabetes (*n* = 115)	Arthritic conditions (*n* = 1107)	Allergies (*n* = 747)	Migraine (*n* = 491)	Neurological diseases (*n* = 27)	Thyroid diseases (*n* = 445)	Vascular diseases (*n* = 536)	Any physical disease (*n* = 2602)
Any specific phobia (*n* = 388)	1.22 (0.88–1.67) (*n* = 69)	1.94 (1.05–3.57)* (*n* = 16)	2.53 (1.73–3.69)* (*n* = 47)	2.20 (1.52–3.16)* (*n* = 49)	1.26 (0.63–2.51) (*n* = 12)	1.49 (1.15–1.92)* (*n* = 131)	1.29 (0.94–1.76) (*n* = 80)	1.99 (1.47–2.69)* (*n* = 78)	0.68 (0.09–4.95) (*n* = 1)	1.72 (1.24–2.37)* (*n* = 69)	1.17 (0.83–1.65) (*n* = 62)	1.87 (1.30–2.68)* (*n* = 289)
Animal subtype^b^ (*n* = 68)	0.88 (0.39–1.97) (*n* = 8)	2.76 (0.82–9.17) (*n* = 3)	3.53 (1.73–7.19)* (*n* = 14)	2.52 (1.10–5.76)* (*n* = 8)	1.92 (0.45–8.15) (*n* = 2)	0.93 (0.48–1.78) (*n* = 15)	2.02 (1.008–4.05)* (*n* = 18)	1.54 (0.79–2.99) (*n* = 13)	4.07 (0.52–31.37) (*n* = 1)	1.44 (0.73–2.86) (*n* = 13)	1.04 (0.49–2.20) (*n* = 10)	1.24 (0.53-2.87) (*n* = 48)
Natural subtype^b^ (*n* = 75)	1.45 (0.75–2.80) (*n* = 15)	2.28 (0.76–6.87) (*n* = 4)	2.53 (0.99–6.41) (*n* = 6)	1.66 (0.62–4.38) (*n* = 6)	0.86 (0.19-3.81) (*n* = 2)	1.55 (0.90–2.65) (*n* = 26)	1.28 (0.61–2.65) (*n* = 13)	2.40 (1.29–4.45)* (*n* = 17)	−^a^	1.41 (0.70–2.80) (*n* = 13)	1.74 (0.89–3.40) (*n* = 16)	2.02 (0.99–4.09) (*n* = 58)
Blood-injection subtype^b^ (*n* = 63)	0.89 (0.38–2.06) (*n* = 8)	2.05 (0.47–8.80) (*n* = 2)	2.20 (0.89–5.39) (*n* = 6)	2.14 (1.05-4.36)* (*n* = 10)	1.03 (0.14–7.49) (*n* = 1)	1.25 (0.68–2.25) (*n* = 19)	0.84 (0.40–1.73) (*n* = 9)	1.60 (0.71–3.55) (*n* = 8)	−^a^	1.56 (0.64–3.73) (*n* = 7)	0.75 (0.30–1.85) (*n* = 6)	0.99 (0.50–1.95) (*n* = 35)
Situational subtype^b^ (*n* = 88)	1.49 (0.86–2.56) (*n* = 22)	1.81 (0.64–5.14) (*n* = 5)	2.35 (1.14–4.85)* (*n* = 10)	1.63 (0.77–3.44) (*n* = 9)	1.17 (0.40–3.44) (*n* = 4)	1.82 (1.12–2.93)* (*n* = 34)	0.84 (0.46-1.52) (*n* = 17)	2.08 (1.16–3.70)* (*n* = 18)	−^a^	2.55 (1.42–4.57)* (*n* = 21)	0.71 (0.36–1.37) (*n* = 13)	1.70 (0.76-3.80) (*n* = 69)
Other^b^ (*n* = 3)	−^a^	−^a^	−^a^	−^a^	−^a^	−^a^	−^a^	−^a^	−^a^	29.91 (2.36-378.42)* (*n* = 2)	−^a^	1.55 (0.12–19.62) (*n* = 2)

*Note.CI* confidence interval, *DSM-IV* Diagnostic and Statistical Manual of Mental Disorders, Fourth Edition, *NOS* not other specified, *OR* Odds Ratio, *n* unweighted number of subjects, *%* weighted percentage, *p < 0.05, ^a^empty cell size, ^b^Subjects did not fulfill the criteria of any other subtype, adjusted for sex

Further, no significant interactions between sex and specific phobia on the association with physical diseases were established. Therefore associations do not differ among men and women.

Among subtypes, the situational subtype was associated with the most (4 of 10) physical diseases, whereas only one association was found between the natural subtype (migraine, OR = 2.40, 95 % CI [1.29–4.45]) and the blood-injection subtype (respiratory diseases, OR = 2.14, 95 % CI [1.05–4.36]) and physical diseases. Gastrointestinal diseases, respiratory diseases, migraine, and thyroid diseases were all associated with two subtypes.

### Age of onset

As shown in Table 3, in the GHS-MHS across all physical diseases most comorbid cases had the onset of specific phobia before the onset of physical diseases.

**Table 3:** Temporal sequence of age of onset of specific phobia and physical diseases among those with comorbidity in the GHS-MHS

Physical disease	Comorbid cases where specific phobia preceded physical condition (%)
Hypertension	82.1
Cardiac Diseases	78.1
Gastrointestinal Diseases	82.0
Respiratory Diseases	80.3
Diabetes	93.0
Arthritic Conditions	77.4
Allergies	73.7
Migraine	72.2
Neurological	0^a^
Thyroid Diseases	79.2
Vascular Diseases	72.3

^a^ *N* = 1

### Replication analysis

In the CoLaus/PsyCoLaus Study, any specific phobia was associated with gastrointestinal, respiratory, arthritic conditions, allergic diseases and migraine (ORs ranging between 1.30, 95 % CI [1.04–1.63], for arthritic conditions and 1.68, 95 % CI [1.33–2.13], for migraine). Further, having any specific phobia was associated with having any physical disease (OR = 1.34, 95 % CI [1.07–1.68]). In this sample, Hypotheses 1–3 could be confirmed. As no associations between specific phobia and cardiac and vascular diseases could be established, Hypotheses 4 and 5 were rejected.

Among subtypes, the animal and natural subtype were associated with respiratory diseases (animal: OR = 1.62, 95 % CI [1.02–2.55]; natural: OR = 2.36, 95 % CI [1.16–4.82]) and migraine (animal: OR = 1.71, 95 % CI [1.09–2.69]; natural: OR = 2.38, 95 % CI [1.16–4.85]). Additionally, the natural subtype was associated with having any physical disease (OR = 3.03, 95 % CI [1.06–8.57]; No other associations were found between subtypes of specific phobia and physical diseases in the PsyCoLaus Study. Results of the PsyCoLaus data are available on request.

Further, similar to the GHS-MHS data across all physical diseases most comorbid cases had the onset of specific phobia before the onset of physical diseases (results available on request).

## Discussion

The aim of our study was to determine the association between specific phobia and its subtypes and physical diseases in a general population sample to extend information from earlier studies by using improved methods (physician-diagnosed physical diseases, standardized diagnostics of specific phobia, a wide range of physical diseases assessed within one sample). Our study suggests an association of specific phobia and several physical diseases in the community. In line with earlier studies, we found an association between specific phobia and respiratory diseases [11, 53], heart diseases [16, 17], vascular diseases [22] and cardiac diseases [23]. An association with gastrointestinal diseases has also been found, but in comparison to our study the association did not remain significant after adjusting for sex [15]. Additionally, we established an association between specific phobia and arthritic conditions, migraine, and thyroid diseases. We could replicate the associations between any specific phobia and gastrointestinal diseases, respiratory diseases, arthritic conditions and migraine in the PsyCoLaus Study. Associations with these physical diseases have already been documented within the GHS-MHS for the group of anxiety disorders [11]. Further, our analyses did not provide evidence for associations between specific phobia and vascular diseases in both data sets. This is not in line with an earlier community based study that established associations between specific phobia and cardiovascular diseases [22]. While we distinguished between cardiac and vascular diseases in two groups as shown in Table 1, the study by Goodwin et al. (2009) only used one group of cardiovascular diseases. Therefore it may be possible that the differences in the established associations may be due to methodological differences.

In addition, even though previous research showed that there are gender differences among specific phobia subtypes [31] and among certain physical diseases such as cardiac diseases [54], we did not establish differences in the associations between specific phobia subtypes and physical diseases among men and women.

Different models can be considered for the explanation of the comorbidity of specific phobia and physical diseases (specific phobia may be an antecedent or a consequent factor of a specific physical disease; there may be common biological or social factors that increase the risk of having both specific phobia and physical diseases; or a third variable may contribute to the comorbidity through an indirect mechanism). Our age at onset analyses show that in most cases specific phobia preceded the physical diseases. Therefore our data suggest that the most probable model at the moment is that a preceding specific phobia increases the risk of subsequent physical disease. Further it may be that a third variable contributes to the comorbidity through an indirect mechanism. The other sequence – a preceding physical disease and a subsequent specific phobia – is the least probable model based on our data. Such analyses can contribute to the deduction of possible etiological pathways of both specific phobia and physical diseases.

It has been suggested that certain pathophysiological mechanisms related to affective and anxiety disorders, such as sympathoadrenal hyperactivity, reduced heart-rate variability, heightened platelet activity, and endothelial dysfunction, may be of interest in the etiology of heart diseases, too [23]. Additionally, ventricular arrhythmia and hyperventilation causing coronary spasm have been named as possible mechanisms by which phobic anxiety and therefore specific phobia cause coronary heart disease [16, 55].

As a plausible explanation for the comorbidity of gastrointestinal and anxiety disorders it has been suggested that anxiety could lead to an irritation of the gastrointestinal system, leading to a gastrointestinal disease [56]. It has also been shown that the neurotransmitter serotonin, which is implicated in anxiety disorders in general and specifically in specific phobia [57], is also known to affect the gastrointestinal system [58].

It has been suggested that anxiety may lead to hyperventilation which is associated with asthma, too [59]. Further, it has been discussed that shared etiological factors (either environmental or genetic) of some anxiety disorders and asthma may account for the comorbidity [60].

Arthritic conditions have mainly been associated with depression, but anxiety disorders seem to be rather common in subjects with arthritis, too [61]. For the evaluation of possible shared etiological factors the following may be considered: certain cytokines and immunglobulins that seem to play a role in arthritic conditions may be associated with mental disorders, too [62].

Several studies have discussed the association of migraine and psychopathology [20, 63, 64]. Psychosocial factors related to stress and therefore specific phobia can act as a trigger for migraine [20]. Additionally, a diminished serotonin level, which is also associated with anxiety disorders, has been detected in subjects with migraine [63]. Further, psychosocial factors such as avoidance of certain situations during anxiety may trigger migraine [63].

An increased rate of subjects with specific phobia has been documented in thyroid disease compared to subjects in the general population [65, 66]. Common neurochemical abnormalities have been identified as a possible link between anxiety and thyroid disease [65].

Beside the association between specific phobia and physical diseases, different patterns of associations between the subtypes of specific phobia and physical diseases have been detected. Most associations were found between the situational subtype and physical diseases. This is in line with a finding from an earlier study that situational phobia has higher rates of comorbid psychopathology than animal and natural phobias [67]. Another study found more somatic symptoms in subjects with natural phobia compared to subjects with animal phobia [32], which might suggest a somatic vulnerability of the natural subtype. In our study, however, the natural subtype was only associated with one physical disease.

The different patterns of associations between the subtypes and physical diseases may point toward different etiological mechanisms that need to be addressed in future studies. As blood-injection phobia is—alone of the subtypes—associated with vasovagal fainting [31], it could be, for instance, that people with this phobia profit from their rather low blood pressure, leading to a reduction of their risk for certain physical ailments, such as cardiovascular diseases or hypertension. Unfortunately, power issues precluded evaluating the impact of blood pressure on the association of specific phobia and cardiovascular diseases. This should therefore be evaluated in future studies.

The current study has a number of limitations. First, the subjects assessed were between 18 and 65 years of age. Therefore the results cannot be generalized to younger or older subjects. Second, some diagnoses may rely more on self-report data (e.g. arthritis) than other (e.g. diabetes) [11]. This could lead to over-reporting of certain symptoms in subjects with specific phobia. Third, no conclusions concerning the causal nature of the associations between specific phobia and physical diseases can be drawn. Longitudinal evaluation of the associations found is needed. Fourth, although the sample size is 4181 subjects, the combination of physical diseases and specific phobia led to relatively small cell sizes, especially in the subtype analyses in the GHS-MHS. Fifth, the comparability of the GHS-MHS and PsyCoLaus study was limited with respect to methodology used and data collected, as different timeframes (12 months vs. lifetime diagnosis of physical diseases) and different age ranges of subjects (18–65 vs. 35–66 years) were used. Further, not all physical diseases were assessed in both studies. Accordingly, it was not surprising that the results from the GHS-MHS study could be partially replicated. Several associations were only observed in the GHS-MHS study, which could be attributable to the younger age of the sample. Indeed, in the older CoLaus/PsyCoLaus sample the prevalence of almost all physical diseases was considerably higher than in the GHS-MHS study and it is therefore possible that an increased number of age related factors predisposing to these diseases could have blurred their association with specific phobia in CoLaus/PsyCoLaus.

Despite these limitations, our study shows that specific phobia is highly comorbid with different physical diseases. Moreover, the different subtypes show different patterns of comorbidity with physical diseases in a community sample.

The comorbidity of specific phobia and physical diseases has been rarely evaluated until now. However, the findings in our community sample support the importance of further research within this field, as the comorbidity may influence the burden of subjects affected [13, 14]. Additionally, this knowledge may be highly relevant for health care policy as studies showed that anxiety disorders may be associated with noncompliance [27] or worse treatment outcome of the physical disease [26]. This means that therapy of anxiety symptoms may not only lead to a reduction of anxiety but also to a better therapy outcome of the physical disease.

Last but not least given that our cross-sectional associations are proved in a longitudinal model and we presume a causal relationship between specific phobia and the different physical diseases this may also have important implications for prevention. Therapy of specific phobia may then reduce the probability of specific physical diseases.

## Conclusions

Our community study shows that specific phobia is associated with an increased probability of specific physical diseases and that the subtypes may differ in their comorbidity patterns with physical diseases. These findings are highly relevant for etiological considerations and crucial for public health aspects, such as treatment, and prevention of the comorbid conditions.

### Availability of data and materials

As the data of the GHS-MHS were obtained from a third party, namely the Robert Koch Institute, we cannot make the data available. However, data from the GHS-MHS are available as a Public Use File from Dr. Frank Jacobi, Institute of Clinical Psychology and Psychotherapy, Chemnitzer Str. 46, 01187 Dresden, Germany; e-mail: jacobi@psychologie.tu-dresden.de. For further information about the GHS Core Survey and its Public Use File, contact Dr. Heribert Stolzenberg, Robert Koch Institute, Nordufer 20, 13353 Berlin, Germany; e-mail: stolzenberg@rki.de. Due to legal reasons it is not possible to make the data of the PsyCoLaus Study available.

## Abbreviations

DIGS: Diagnostic Interview for Genetic Studies
DSM: Diagnostic and Statistical Manual of Mental Disorders
GAD: Generalized Anxiety Disorder
GHS-MHS: German Health Interview and Examination Survey, Mental Health Supplement
ICD: International Classification of Diseases
M-CIDI/DIA-X: Munich Composite International Diagnostic Interview
SADS-LA: Schedule for Affective Disorders and Schizophrenia – Lifetime Version
